# Generating Causal Relations in Scientific Texts: The Long-Term Advantages of Successful Generation

**DOI:** 10.3389/fpsyg.2019.00199

**Published:** 2019-02-05

**Authors:** Roman Abel, Martin Hänze

**Affiliations:** Department of Educational Psychology, University of Kassel, Kassel, Germany

**Keywords:** coherence, causal cohesion, expository text, generation effect, aptitude-treatment interaction, cognitive load, dual-task, desirable difficulties

## Abstract

A high level of text comprehension can be achieved by engaging learners in processes of organization and integration while reading a cohesive text. In the present study, we investigated the impact of an innovative generative technique on learning with scientific texts. The *cohesion generation* was implemented by means of *explicit* cohesion gaps. High school students (*n* = 199) were randomly assigned to either receive a fully cohesive scientific text (control condition) or a scientific text that required the selection of causal connectives, such as *because*, *although*, *therefore*, or *however* (generation condition). Learners in the generation condition were required to reflect on causal relations to complete the text. All students were tested immediately (T1) and 2 weeks after the learning phase (T2). Cognitive load was measured by a dual task and self-report measure. Contrary to our expectations, no differences were found in performance on inference questions (situation model). Learners in the generation condition performed worse on text-based questions at T1 but showed less forgetting from T1 to T2. The impact of condition on the situation model was moderated by reading skills. Remarkably, the generation success was highly predictive for learning outcomes even when controlling for learners’ proficiencies. Consequently, learners who succeeded to employ effortful processes to overcome the difficulty showed a superior performance on both the text-base and situation-model questions compared to students reading the cohesive text. Moreover, in these learners, generative activity led to a sustainable learning performance 2 weeks later. Poor readers especially took advantage of generative activity, despite struggling to perform the cohesion task as indicated by the cognitive load measures. The results suggest that the activity of generating causal relations can augment inferential processing in learners who are not involved in inferential processing spontaneously. To successfully apply this generative learning technique, students require considerable instructional support.

## Introduction

Expository texts are a major source of scientific knowledge in educational settings. Unexperienced readers, however, struggle with expository texts, because the content in general and the macrostructures of the text are usually unfamiliar to them (Meyer, 1975; Cook and Mayer, 1988; Lorch, 2015). Apart from the complexity and informational density of scientific texts, the multi-causality of scientific phenomena appears to be especially challenging for readers (cf. Britt et al., 2014). Accordingly, learners have difficulties selecting the main ideas from the text, organizing them in a meaningful way, and integrating the content with previous knowledge. As reading of expository texts rarely goes beyond a shallow text-based representation, learners fail to construct a coherent representation (situation model) of the learning content. Poor readers may especially struggle to understand the content from scientific texts. As opposed to skilled readers, poor readers have difficulties in bridging inferences from distant idea units in the text and integrating novel content with previous knowledge (Hannon and Daneman, 2001), which are essential processes for the situation-model construction (Kintsch, 1988). Thus, one very important aim of instructional science in general and of this study in particular is to provide recommendations on how to increase the readability of expository texts and to facilitate the processes of knowledge construction during reading.

### The Gap Between Cohesion and Coherence

In short, there are two ways to promote learning from expository texts. The first way is to provide learners with a well-written text. The research on reading comprehension has identified several text characteristics that make the text easier to understand (Graesser et al., 2011). Among other characteristics, causal cohesion is considered an essential characteristic for supporting the coherence formation (Noordman and Vonk, 1997; Sanders and Noordman, 2000; Louwerse, 2001). A text can be regarded as causally cohesive if the causal relations between propositions, clauses, and sentences are explicitly marked by connectives, such as *because*, *therefore*, *however*, and *although*. These linguistic markers provide readers with explicit instructions for organizing adjacent and distant concepts from the text into a network of relations (Gernsbacher, 1990; Zwaan and Radvansky, 1998). Moreover, to validate the causal relations encountered in the text, readers make world knowledge inferences by retrieving general premises (Noordman et al., 1992). Thus, connectives support the integration of new content with previous knowledge. When learners lack the necessary knowledge, general premises can be inferred and assimilated into their knowledge base (Cozijn et al., 2011). Numerous studies have demonstrated the positive impact of cohesion devices on the memory of causally connected sentences compared to isolated sentences (Trabasso and van den Broek, 1985; cf. Myers et al., 1987; Fletcher and Bloom, 1988) and on reading comprehension (Degand et al., 1999; Linderholm et al., 2000; Degand and Sanders, 2002; Maury and Teisserenc, 2005; Sanders et al., 2007; Van Silfhout et al., 2014a,b, 2015).

The second way to promote learning from expository texts is by directly engaging learners in active knowledge construction. For example, encouraging learners to self-explain while reading prompts them to draw inferences, monitor their own understanding, and detect and repair the flaws in their mental representation (for a review, see Wylie and Chi, 2014).

Engaging students in active knowledge construction with poorly written texts or engaging them with a cohesive text deprived of active processing provides an insufficient basis for establishing deep comprehension. Apparently, incorporating both cohesion and active processing is necessary to optimize learning. Ainsworth and Burcham (2007) showed that self-explanation from a maximally cohesive text leads to superior comprehension compared to self-explanation from minimally cohesive text. Thus, the function of self-explanation seems to change depending on information provided by the text structure. In minimally cohesive texts, self-explanation serves to compensate for the cohesion gaps, whereas in fully cohesive texts, self-explanation supports the coherence formation based on explicit relations. This finding supports the view that cohesion and generative learning address different aspects of knowledge construction. Both processes appear to be necessary for coherence formation. Active processing should be promoted to establish a congruent relation, whereas linguistic markers should be used to provide the instruction of *how* to relate information. Following this reasoning, active processing of minimally cohesive texts may result in efforts unconnected to schema construction.

Correspondingly, a fully cohesive text itself does not sufficiently initiate coherence formation and often leads to shallow processing. For example, Millis et al. (1993) found no retention benefit for causally connected statements. Noordman et al. (1992) found that readers did not spontaneously construct inferences of unfamiliar causally related clauses. Instead, the level of active processing depended on how the reader made use of the information. Only those readers who were prompted to judge for inconsistencies or to respond to questions about a causal relation in the text generated inferences. Thus, reading processes heavily depend on learners’ goals and the nature of the reading task (Graesser et al., 2015).

According to the minimalist hypothesis, reading a locally cohesive text does not result in the generation of global inferences. In contrast, inconsistencies and disruptions on the local level compel readers to draw inferences to fill the gaps (McKoon and Ratcliff, 1992). Reading a well-written text can even result in a decrease of coherence formation in high prior-knowledge learners because well-written texts do not require readers to make inferences (McNamara and Kintsch, 1996; McNamara et al., 1996). In line with this finding, Schworm and Renkl (2006) reported a decrease in quality of self-generated explanations when instructional explanations were provided for learners.

In the present article we address the following problem: a minimally cohesive text promotes processes of coherence formation but does not provide the necessary instructions for how to establish coherence, whereas a fully cohesive text provides the instructions for how to establish coherence but lowers the necessity to do so. These considerations underscore an open gap between cohesion as a text characteristic and coherence as the situation model of text content. Consequently, this article addresses the research question of how to close the gap between cohesion and coherence construction when reading expository texts. For this purpose, we designed a cohesion generation task that was intended to engage learners in coherence construction while reading.

### Benefits and Costs of Generative Learning

A learning advantage of reading strategies that require active processing, compared to passive approaches such as restudying, is called the *generation effect*. According to Wittrock’s (1989) generative model of learning, the generation effect is due to the *internal* connections learners build between the information units of the to-be-learned materials and the *external* connections learners build between new content and previous knowledge. The internal and external connections as specified in Wittrock’s (1989) model of generative learning can be compared to the central ingredients of further prominent models of meaningful learning, such as the processes of *construction* and *integration* within the *CI* framework (Kintsch, 1988) or the processes of organization and integration within the *select-organize-integrate* (SOI) framework (Mayer, 2014).

The classic experiments on the generation effect (Jacoby, 1978; Slamecka and Graf, 1978) entailed a large body of research on the generation of simple word associates (for a meta-analysis, see Bertsch et al., 2007; for a review, see McNamara, 1992). In these and similar experiments, learners in the generation condition were presented with incomplete words that needed to be completed according to specific rules. The generative activity of learners engaged them in more effortful processing compared to simply reading, and therefore increased long-term retention. Thus, challenging learners may be regarded as a desirable difficulty (cf. Bjork and Bjork, 2014). However, the insights from studies on generative learning that have employed only word associates in their design are not applicable for educational practice for numerous reasons. A word-completion task does not necessarily involve learners in relational processing nor lead to deep comprehension (McDaniel and Butler, 2011). According to cognitive load theory (CLT), element “interactivity,” as defined by CLT, is very low in the case of word lists because the elements can be processed in isolation (Sweller, 2010). Consequently, demands of processing such learning materials are very low. Given the low-complexity of learning materials used in studies on generative learning, the examination of learning outcomes was limited to simple retention. Thus, whether generative activity while studying complex and coherent materials benefits learning remains controversial (Chen et al., 2015, 2016). The gains from generative learning in terms of promoting relational processing might be outweighed by the costs of overwhelming learners.

Research on generative learning with complex and coherent materials, such as expository texts, widened the range of learning outcomes toward deep comprehension and transfer of novel knowledge. Additionally, generation activity diverged to particular generation targets (i.e., *what to generate*) and the kind of implementation by the type of task (i.e., *how to generate*). A few prominent generative learning strategies emerged from this research, such as the generation of concept maps (Nesbit and Adesope, 2006), drawings (Leutner and Schmeck, 2014), text structure via sentence scrambling (McDaniel and Butler, 2011), questions (Song, 2016), elaborative interrogations (Seifert, 1994), and self-explanations (Wylie and Chi, 2014). All generation approaches have in common that the to-be-generated units of information should be inferred based on the text rather than retrieved directly from the text (Fiorella and Mayer, 2016). For example, during the drawing activity, learners are required to transform the textual information into a visual representation (Leutner and Schmeck, 2014). In the case of self-explanation, the explanations should elaborate beyond the explicitly provided textual information (Wylie and Chi, 2014). Generation prompts serve the function of either supplementing the elaboration on complete learning materials (e.g., self-explanation during reading, or concept mapping after reading) or completing the initial learning material (e.g., word-completion task or scrambling sentences).

Along with the increased focus on learning material complexity, the consideration of generation success also became important. Successful learning is assumed to be contingent on the accuracy of generation task performance. Thus, learners must be able to perform the generation task accurately to unfold the potential of generative learning. However, most students are barely instructed to use generative learning strategies in educational settings. Given the lack of opportunities to practice generative learning during education, it may not be surprising that students usually process learning content passively and use learning strategies that target only rote learning (cf. Dunlosky et al., 2013). The advantages of generative learning may even be reversed, because learners gain a considerably higher expertise in passive learning strategies, such as restudying. According to the *randomness as genesis principle*, unsupported generation imposes a high level of extraneous cognitive load on learners’ working memory, which consumes cognitive resources that as a consequence are no longer available for schema construction (Paas and Sweller, 2014; Chen et al., 2015, 2016). Furthermore, learners are not accustomed to performing generative strategies, thus their proficiencies, such as reading skill, previous knowledge, or general intelligence, may substantially contribute to generation success in particular and learning in general. Several studies on generative learning have shown greater advantages of generation on learning when subjects received merely a short-term training on how to perform the generation task (e.g., for drawing, see Leopold, 2009; for summarization, see Friend, 2001; for concept mapping, see Holley et al., 1979; for self-explanation, see McNamara, 2004; McNamara et al., 2006). Hence, successful learning depends on promoting and supporting active processing.

### Generation of Causal Relations

A growing body of evidence from research on generative learning suggests that elaborating on causal relations supports coherence formation. For example, Allen et al. (2015) found a link between the learning performance and the extent of causal cohesion in students’ language responses during self-explanation and think-aloud activities. Kurby et al. (2012) also found that local and distal inferences during self-explanation predicted comprehension. Similarly, Magliano and Millis (2003) demonstrated that readers whose verbal protocols overlapped with causally important sentences from the text achieved higher scores on the comprehension test.

The importance of reflecting on causal relations is broadly acknowledged in the research on generative learning. Generative learning strategies, such as elaborative interrogation, question generation, or concept mapping, likely entail deep processing because of the reflection on factual statements in terms of causes and consequences or reasons and claims. For example, the studies on elaborative interrogation showed that why-prompts promote learners to reflect on reasons, conditions, and causes of certain facts (cf. McDaniel and Donnelly, 1996; Ozgungor and Guthrie, 2004; Smith et al., 2010). Similarly, generating high-level questions, which target conceptual and causal relations in text, supports comprehension (Bugg and McDaniel, 2012). Engaging students in learning with concept maps triggers them to analyze the learning content in terms of causes and consequences (McCrudden et al., 2007). Moreover, theoretical underpinnings and a large body of empirical evidence exists for considering *deep comprehension* as a highly interconnected representation (Zwaan and Radvansky, 1998). Experts, as opposed to novices, possess a sophisticated network-representation of causes and consequences in their knowledge domain (Noordman et al., 2000). Accordingly, a powerful generative learning strategy ought to direct learners’ focus on causal relations among factual statements in the learning content.

Participants in our study were required to generate causal relations between factual statements in a text in which causal connectives were removed, leaving behind visible gaps. Arguably, the absence of linguistic markers do not automatically promote the processes of organization and integration. Readers need to be aware of the cohesion gaps to close them (Glaser, 1989), but they often miss the *implicit* cohesion gaps in texts. Numerous studies have attributed the inferiority of poorly written texts to learners’ inability to close cohesion gaps (McNamara and Kintsch, 1996; McNamara et al., 1996; Kamalski et al., 2008). However, the lack of ability to detect cohesion gaps has yet to be explored as an alternative explanation. Thus, the demands imposed by reading a minimally cohesive text may be additionally attributed to the detection of cohesion gaps. In light of this view, the superiority of self-explaining while reading fully cohesive compared to minimally cohesive texts in the study of Ainsworth and Burcham (2007) can be partially attributed to additional demands that were imposed by *implicit* gaps. In contrast, the cohesion gaps in our study were *explicitly* marked as gaps in the text, and the generation activity was explicitly required for these gaps. We investigated the extent that a cohesion generation task during reading can facilitate construction and integration processes.

### Present Study

The generative learning technique we used extends the existing variety of generation techniques. Learners in the generation condition read text in which conjunction gaps were placed, and they were instructed to establish a causal relation for each gap by choosing the appropriate connective between four alternatives, *because*, *although*, *therefore*, or *however*. These connectives indicated causal relations between clauses, and varied systematically in *polarity*—positive vs. negative—and *direction*—backward vs. forward (cf. taxonomy reported in Sanders et al., 1992; Louwerse, 2001). *Positive* (*because, therefore*) vs. *negative* (*although, however*) refers to confirming vs. violating expectations (Lagerwerf, 1998). The expectation is explicitly conveyed in positive-polarity sentences, whereas negative causal relations add a contrastive meaning to the given causal link. *Backward* (*because*, *although*) vs. *forward* (*therefore*, *however*) refers to the direction of cause and consequence. A backward connective heads the cause, whereas a forward connective is followed by the consequence. Thus, to choose the correct connective, learners were required to indicate the direction (*What is the cause and what is the consequence?*) and polarity (*Are the cause and consequence intuitive or counterintuitive?*). In contrast, without the need to evaluate causal relations while reading a fully cohesive text, the clauses within a sentence might simply be accepted by the readers as being causally related (Cozijn et al., 2011). Accordingly, the generation of causal relations was intended to bridge the gap from cohesion to coherence formation when reading an expository text.

The study was conducted in a German high school. Thus, the text was written in German using German counterparts of causal connectives: *weil* (because), *obwohl* (although), *deswegen* (therefore), and *dennoch* (however). One limitation of using the German language when employing a cohesion generation task should be noted. The direction of connectives and syntax are confounded. The German grammar rules of sentence construction change depending on the connective. The verb in the second clause must be placed next to the connectives *deswegen* and *dennoch* (forward direction), whereas the verb in the second clause must be placed at the end of the sentence when the connectives *weil* and *obwohl* (backward direction) are used. Consequently, the direction can be derived based on the position of the verb. Hence, generation choices could be partially made based on syntactically driven conclusions.

In our study, each of the to-be-generated target words was embedded between two clauses within a sentence. The choice of target word was based on the meaning of contextual information. For example, the choice between *because* or *therefore* completely depends on the meaning of the preceding and subsequent clauses. Few published studies have used the word-generation task when expository statements are read (e.g., Peynircioglu and Mungan, 1993; DeWinstanley and Bjork, 2004). However, in the study of DeWinstanley and Bjork (2004), learners were only required to fill in the missing letters of target words. Consequently, the task could be performed nearly independently from the contextual information. In the study of Peynircioglu and Mungan (1993), participants were required to recall words during the final test that they had generated during the learning phase. In contrast, the cohesion generation task in the present study was intended to promote the learning of complex information in the surrounding text.

Given that the advantages of generative learning may be attributed to the processes of organization and integration, we were particularly interested in capturing indices of inferential processing during the generative activity. Thus, along with learning outcomes, we assessed online processing measures such as time-on task, generation success, and cognitive load per self-report and via a dual task.

Generative learning—as claimed by the desirable difficulty framework—may lead to a subjective experience of a more effortful processing but also to long-term advantages in learning. Accordingly, participants in the generation condition were expected to experience a higher cognitive load caused by additional inferential processing and to achieve higher test scores after a 2-week delay.

### Hypotheses

The generation task targeted the comprehension of relations between the concepts by requiring learners to infer the causal relations between the clauses. Based on the distinction of different levels of information integration in the *CI* framework (Kintsch, 1988), we expected the participants in the generation condition to benefit primarily in terms of the *situation model* assessed by high-level inference questions (H1). Answering such questions requires learners to relate multiple idea units from the text (organization) and to integrate the novel content into a coherent representation. Moreover, the *text-based* representation—assessed by low-level retention questions—also might be promoted (H2), because learners need to reprocess and reflect on the meaning of the previous and successive clauses to establish a causal relation. Information necessary to answer text-based questions can be simply recalled from the memory of single sentences. We also expected a long-term advantage of generative learning in terms of lower forgetting rates (H3).

The level of difficulty can hamper learning. We therefore considered several aspects and restrictions in generative learning. The generation effect on the situation model formation might depend on a successful inference of causal relations during the generation activity. A low accuracy reflects a failed attempt of constructing an appropriate representation of relationships, whereas a high generation accuracy indicates a coherent mental model. Therefore, we expected that only learners who perform accurately on the generation task could take advantage of the generation activity in terms of situation model construction (H4). In contrast, the text-based representation might depend less on generation success, because a low-level question targets the retention of isolated sentences rather than inferences. Thus, we expected to find a generation benefit even in students who showed a low performance during the generation task.

We further assumed that generation success would strongly depend on learners’ proficiencies such as reading skills. Results from studies on reading comprehension have suggested that reading skill is an important factor in learning from complex expository texts. Its impact on learning is independent from previous knowledge (Voss and Silfies, 1996; O’Reilly and McNamara, 2007; Ozuru et al., 2009). The importance of strategic processing increases when learners lack the knowledge needed to bridge cohesion gaps in complex scientific texts (Lorch, 2015). Reading skills help learners to relate multiple ideas and various concepts throughout a text via effortful inferential processing and integrate textual information in a coherent mental representation (Hannon and Daneman, 2001). However, we expected that skilled readers would not benefit from the generation activity as much as the poor readers. The generation activity might be redundant in skilled readers, because a high level of reading skill is associated with spontaneous inferential processing during reading. In contrast, less skilled readers might lack the spontaneous use of inferential processing (McDaniel et al., 2002). Thus, an explicit instruction to generate causal relations might engage poor readers in organization and integration processes and in turn promote learning (H5).

## Materials and Methods

### Design

Participants were randomly assigned to one of two learning conditions. In the control condition, participants read a cohesive text in which the clauses in the text were explicitly linked by means of causal connectives. In the generation condition, the text lacked the connectives. Learners were then instructed to choose between four alternatives—the German counterparts of *because*, *although*, *therefore*, or *however*—from a dropdown list for each missing link. See Table 1 for a direct comparison in which an exemplary paragraph from the control and the generation conditions are juxtaposed. The retrieval interval was manipulated as a within-participants factor, testing participants immediately and after a 2-week delay. We tested students’ text retention and comprehension.

**Table 1:** A sample text paragraph taken from the control and generation condition for comparison.

High causal cohesion (control)	Generation of causal cohesion
Solar radiation can be absorbed by the Earth’s land surface and stored as heat, however, some sunrays partially rebound. Reflection can happen on any surface, although some surfaces seem to be unsuitable. In certain cases, this phenomenon is called specular reflection, because the angle of reflection equals the angle of incidence. Diffuse reflection refers to the case that the incident ray is evenly reflected at many angles. If the incident ray is unevenly reflected at many angles, the phenomenon is called mixed reflection. Nature offers a variety of rough surfaces, therefore the mixed reflection is the most common case. A part of sunrays, which have been reflected, do not lose any energy, therefore its waves remain short. The reflected sunrays pass the atmosphere without being absorbed and escape into space because they retain short waves.	Solar radiation can be absorbed by the Earth’s land surface and stored as heat, __________ some sunrays partially rebound. Reflection can happen on any surface, __________ some surfaces seem to be unsuitable. In certain cases, this phenomenon is called specular reflection, __________ the angle of reflection equals the angle of incidence. Diffuse reflection refers to the case that the incident ray is evenly reflected at many angles. If the incident ray is unevenly reflected at many angles, the phenomenon is called mixed reflection. Nature offers a variety of rough surfaces, __________ the mixed reflection is the most common case. A part of sunrays, which have been reflected, do not lose any energy, __________ its waves remain short. The reflected sunrays pass the atmosphere without being absorbed and escape into space, __________ they retain short waves.


*The text was translated from German. The translation into English lacks the syntactical hints on the direction of causal relations that are necessary in German (see Present Study). In the generation condition, the text lacked the connectives. Learners were instructed to click on the missing links to choose the correct connective from a dropdown list.*

### Sample

In total, 199 German high school students (grades 10–12) participated in the experiment of which 112 students were randomly assigned to the cohesive text condition and 87 students to the generation condition. Of the 199 students, 21 students were absent during the second examination (12 students in the cohesive text condition and 9 students in the generation condition). On average, students were 18 years old (*M* = 17.7; *SD* = 2.3), 44.7% were female, and 33.1% reported another native language instead of or besides German. The study was conducted during a regular class lesson. Students studied individually with notebooks. We received written informed parental consent for all participants under 18 in accordance with the Declaration of Helsinki.

### Learning Materials

The study was programmed with *Inquisit 3* and presented on a notebook screen. Topics of the scientific text were climate change, global warming, and the greenhouse effect. The text was written in German and comprised 18 passages (124 sentences; 2,089 words in total). Each passage was presented on a slide with a headline above the text. Participants could click on *continue* to skip forward to the next passage, but no option was provided to skip back.

The scientific text was developed specifically for this study. The causal relations between the clauses were experimentally manipulated. In the control condition, participants read a fully cohesive text. In this text version, a total of 57 causal relations were made explicit by the four connectives, *weil* (*because*), *deswegen* (*therefore*), *obwohl* (*although*), and *dennoch* (*however*). The frequency of each connective in the text was different because of the constraints in creating text in which the variation in *polarity* throughout the text is more inflexible than the variation in *direction*. Negative-polarity connectives denote the negation of readers’ expectations. Thus, a negative causal relation presumes the preexistence of such expectations that contradict the real phenomena (Lagerwerf, 1998). The connectives of negative polarity consequently appeared less frequently in the text (*although* = 7; *however* = 8), whereas the connectives of positive polarity appeared more frequently (*because =* 25; *therefore* = 17).

In the generation condition, the missing connective was indicated by a gap in a sentence. Students were instructed to choose one of the four connectives (*because*, *although*, *therefore*, and *however*) from a dropdown list, which could be activated by clicking on the gap. The choice required the participant to infer the connective based on the causal relation between two clauses. After choosing a connective, it was still possible to reconsider the decision and choose again. All gaps within the presented paragraph were required to be completed before advancing to the next page.

For a direct comparison, Table 1 shows sample text passages about the reflection of sunrays for the control and generation conditions. The text was translated from German into English. Note that the English version contains no syntactical hints on causal relations (see Present Study).

### Measures and Scores

#### Learners’ Proficiencies

Reading skill was assessed with the Reading-, Speed,- and Comprehension Test for grades 6–12 by Schneider et al. (2007). According to the manual, students were given 4 min to proceed through the text as far as possible. The simultaneous task was to choose the correct word out of three alternatives for each encountered gap in the text. Given this context, participants were required to select the appropriate term. This measuring instrument was chosen because of the overlapping of cognitive demands with the cohesion generation task.

Previous knowledge on the topic was measured with 16 verification items and two open questions (e.g., *What is the natural greenhouse effect?*). The verbal component of general intelligence was assessed via the word-analogy subtest from the cognitive ability test by Heller and Perleth (2000). This test required that the participants analyze the relation between two presented word stimuli to choose the correct target word out of five alternatives, which is related to a new word stimulus in the same way.

No significant differences were found in the three proficiency measures between the two groups; reading skill *t*(176) = 1.31, *p* = 0.191, previous knowledge *t*(176) = 0.75, *p* = 0.456, and word analogy *t*(176) = 0.64, *p* = 0.523.

#### Learning Processes

The responses on the cohesion generation task were recorded. The individual scores reflected the number of correctly constructed causal relations out of 57 relations in total. High scores indicate a high level of successful relational processing.

Cognitive involvement during reading was assessed by means of the dual task. Reaction time and accuracy of the responses were recorded. Quick and accurate reactions indicate a low load on working memory (Brünken et al., 2002; cf. Park and Brünken, 2015). The dual task required a quick verification response to a trivial mathematical equation, which was either true (e.g., 5 + 1 = 6) or false (e.g., 1 + 1 = 0). A randomly chosen mathematical equation appeared once per slide in a randomly determined moment. Participants were instructed to hold their left hand on the keyboard and press *A* for *false* and *S* for *true* as fast as possible.

To differentiate cognitive load types, a questionnaire developed and evaluated by Leppink et al. (2013) was used and adopted for the current learning material in German. The scale includes 10 items; three items for *intrinsic load* (e.g., *the topic covered in the activity was very complex*), three items for capturing the *extrinsic load* (e.g., *the explanations were, in terms of learning, very ineffective*), and another four items for *germane load* (e.g., *the activity really enhanced my understanding of the topic covered*). The response scale is between 0 (meaning *not the case at all*) and 10 (meaning *completely the case*).

#### Learning Outcomes

The final test consisted of 59 sentence verification tasks, three matching tasks, and three open questions. These questions were designed to assess two different types of knowledge: the text-based representation and the situational model.

The text-based representation was assessed through low-level questions on isolated propositions. The necessary information to answer these questions could be found within single sentences. Text-based questions included 27 verification items and three matching tasks. Students responded to the verification task by choosing whether a statement was true or false. The statements could be recognized based on the explicit information that appeared in the text (e.g., *hot objects emit radiances with a short length* as a true statement; *hydrogen is a greenhouse gas* as a false statement). The matching task required participants to connect detailed information units that belonged together (e.g., *assign the following gasses—oxygen, azote, carbon dioxide, noble gasses—to the concentrations in the atmosphere—78, 0.03, 21, 1%*). Cronbach’s α for the text-based questions were acceptable (immediate testing = 0.79; delayed testing = 0.69).

The situation model was assessed through high-level questions, which required participants to draw inferences from multiple sentences in the presented content. Situation model questions included 32 verification items (e.g., *sun radiances can be reflected on sand* as a true statement; *it gets colder on Earth if the warmth gets absorbed* as a false statement) and three open questions. The open questions assessed conceptual understanding (e.g., “*Please explain how it gets warmer within the greenhouse compared to outside*”). The responses on open questions were scored by two student-assistants depending on the number of main ideas mentioned by the participant. The average interrater reliability was 0.91 for immediate and 0.95 for delayed testing. Discrepancies were resolved by discussion. Cronbach’s α for the situation model questions were 0.76 for immediate testing and 0.73 for delayed testing.

### Procedure

The study was conducted during a regular class lesson. Students studied individually with notebooks. The examination took place on two days with a 2-week delay.

Following the test on previous knowledge, subjects received instructions combined with a training on the dual task. The participants were randomly assigned either to read a cohesive text or to generate the causal cohesion while reading an incomplete text. The students were instructed to read the text carefully to be able to answer the questions in the following test on memory and comprehension. Learners in the generation condition were further instructed on how to perform the generation task and to read carefully to be able to accurately choose the correct connective. While reading the text, a mathematical equation appeared once per text-slide. Students were required to quickly indicate whether the equation was true or false (dual task to objectively measure the cognitive load). When the participants finished reading, they answered questions about their experience of cognitive load. Participants then immediately worked on the final test. In most cases, the examination at T1 took no more than an hour.

The follow-up test was administered 2 weeks later. Participants were tested individually on the computer. They worked on the same questions as 2 weeks earlier. Then, reading skills and word analogy were assessed. The examination at T2 took approximately half an hour.

## Results

### Learning Processes

The generation and control conditions were compared on measures recorded during the learning phase and afterward by computing independent-samples *t*-tests. Means and standard deviations in the time-on task and cognitive load measures are reported in Table 2.

**Table 2:** Mean scores and standard deviations of learning processes.

	Control		Generation	
Measure	*M*	*SD*	*M*	*SD*
Time-on task (in min.)	15.44	5.44	20.16	5.91
Dual-task reaction time (mean in ms)	2414	1925	2311	754
Dual-task accuracy (in %)	94.17	7.83	95.94	6.10
Intrinsic CL	5.59	2.18	5.88	2.45
Extraneous CL	3.18	2.19	3.8	2.09
Germane CL	6.38	2.26	5.75	2.29


#### Time-on Task

Learners in the generation condition spent significantly more time reading the text, indicating a higher involvement because of the generation task, *t*(197) = -5.85, *p* < 0.001.

#### Cognitive Load via Dual Task

The objective measure of cognitive load via a dual task revealed no differences between the two groups in reaction time, *t*(197) = 0.47, *p* = 0.639, and response accuracy, *t*(197) = -1.74, *p* = 0.084.

#### Cognitive Load via Self-Report

The self-report measures of cognitive load were also analyzed. The groups did not differ in their perceived complexity of the text in terms of intrinsic load, *t*(197) = -0.90, *p* = 0.368, and germane load, *t*(197) = 1.93, *p* = 0.055. However, generation activity imposed a significantly higher extraneous load, *t*(197) = -2.05, *p* = 0.043.

### Generation Success

Students, on average, chose the correct connective in three out of four sentences (*M*_%accuracy_ = 73.97; *SD* = 15.81).^1^

#### Correlations With Learning Processes

We focused on three learning processes involved in the generation activity in or investigation of the impact of generation success on learning outcomes. As Table 3 shows, generation success increased the more time participants spent on reading (*r* = 0.47, *p* < 0.001) and the quicker they responded on the dual task (*r* = -0.41, *p* < 0.001). The latter correlation indicated that learners who experienced less restriction on memory capacity could more efficiently employ their cognitive resources for establishing causal relations. This interpretation is supported by the finding that generation success was also associated with a higher level of germane load (*r* = 0.32, *p* = 0.003) and a lower level of extraneous load (*r* = -0.34, *p* = 0.001).

**Table 3:** Pearson correlations between dependent measures.

	1	2	3	4	5	6	7	8	9	10	11	12	13	14
**Learning processes**														
(1) Time-on task		–0.02	0.19**	0.02	0.00	0.12	0.47**	0.09	0.11	0.07	0.20**	–0.07	0.10	0.02
(2) Dual-task RT			0.14	–0.08	0.09	–0.06	–0.41**	–0.15*	–0.05	–0.10	–0.18*	–0.10	–0.11	–0.08
(3) Dual-task accuracy				0.04	–0.19**	0.07	0.07	0.05	0.06	0.16*	0.14	0.11	0.03	0.09
(4) Intrinsic CL					0.34**	–0.10	–0.03	–0.28**	–0.34**	–0.25**	–0.17*	–0.12	–0.27**	–0.20**
(5) Extraneous CL						–0.34**	–0.34**	–0.36**	–0.28**	–0.36**	–0.36**	–0.34**	–0.30**	–0.22**
(6) Germane CL							0.32**	0.34**	0.20**	0.27**	0.33**	0.143	0.18*	0.16*
(7) Generation success								0.73**	0.63**	0.76**	0.76**	0.44**	0.57**	0.61**
**Learning outcomes**														
(8) Text base T1									0.75**	0.74**	0.71**	0.46**	0.61**	0.55**
(9) Text base T2										0.67**	0.68**	0.41**	0.52**	0.58**
(10) Situation model T1											0.74**	0.51**	0.64**	0.60**
(11) Situation model T2												0.46**	0.60**	0.59**
**Learning proficiencies**														
(12) Reading skill													0.36**	0.43**
(13) Prior knowledge														0.49**
(14) Word analogy														


*^∗^*p* < 0.05. ^∗∗^*p* < 0.01. The correlations with the generation success could only be computed in the generation condition. The correlations with the text-based representation on T2, the situation model on T2, reading skill and word analogy was computed only for participants who completed the examination on T1 and T2.*

#### Dependency on Learners’ Proficiencies

We attribute the individual accuracy in generating causal relations to learners’ ability to bridge inferences across isolated ideas in text and to integrate new content into previous knowledge. Thus, the relation between generation success and learners’ proficiencies was particularly interesting. Generation success significantly correlated with reading skills (*r* = 0.44), prior knowledge (*r* = 0.57), and word analogy (*r* = 0.61), all *p-*values < 0.001 (see Table 3). We computed an OLS linear regression with reading skills, prior knowledge, and word analogy as predictor variables, and generation success as a criterion variable. Overall, the model was significant, *F*(3,74) = 32.19, *p* < 0.001, and explained 56.6% of the variance. All three proficiencies were significant predictors of generation success: reading skill [*β* = 0.18, *t*(74) = 2.11, *p* = 0.038], prior knowledge [*β* = 0.42, *t*(74) = 5.05, *p* < 0.001], and word analogy [*β* = 0.37, *t*(74) = 4.15, *p* < 0.001].

#### Impact on Learning Outcomes

Generation success was significantly related to learning outcomes for text-based representation and the situation model at both measurement points (correlations ranged between 0.63 and 0.76, all *p-*values < 0.001). Note that the correlations between learning outcomes and learners’ proficiencies were also significant (correlations ranged between 0.41 and 0.64, all *p-*values < 0.001). The question of interest is whether generation success predicts learning outcomes over and above learners’ proficiencies. We computed a stepwise regression analysis separately for text-based representation on the immediate and delayed final test scores and the situation model on the immediate and delayed final test scores. We entered the three predictor variables, reading skill, prior knowledge, and word analogy in the first step and generation success in the next step. Generation success significantly predicted the learning outcomes over and above learners’ proficiencies: text-based representation T1 [*β* = 0.42, *t*(73) = 3.84, *p* < 0.001, *R^2^* changed from 0.54 to 0.62, *F*(1,73) = 14.78, *p* < 0.001], and T2 [*β* = 0.33, *t*(73) = 2.53, *p* = 0.014, *R^2^* changed from 0.42 to 0.47, *F*(1,73) = 6.38, *p* = 0.014]; and the situation model T1 [*β* = 0.54, *t*(73) = 4.92, *p* < 0.001, *R^2^* changed from 0.50 to 0.62, *F*(1,73) = 24.25, *p* < 0.001]; and T2 [*β* = 0.47, *t*(73) = 4.60, *p* < 0.001, *R^2^* changed from 0.57 to 0.66, *F*(1,73) = 21.17, *p* < 0.001].

### Learning Outcomes Irrespective of the Generation Success

A repeated measures ANCOVA with the condition (cohesive text vs. generation condition) as a between-subjects factor and the delay (immediate vs. 2 weeks delay) as a within-subjects factor was computed for text-based representation and the situation model separately. We included the z-standardized score for reading skills as a covariate in the analysis to control for the effect of learners’ spontaneous relational processing.

#### Text-Based Representation

**FIGURE 1:**
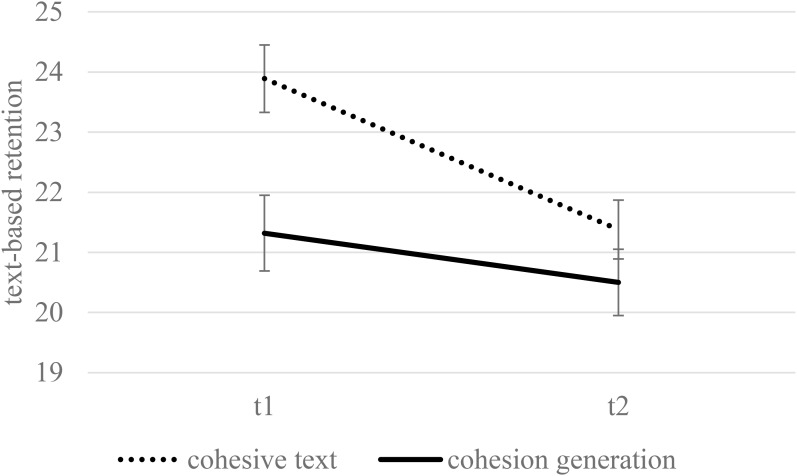
Text based representation as a function of condition and delay when controlling for reading skills (estimated means and standard errors). Maximum performance was 38.

Figure 1 displays the means and standard errors for text-based questions in the final test as a function of condition and retention interval. No significant main effect of condition was found on retention performance collapsed across both tests, *F*(1,174) = 3.73, *p* = 0.055, η^2^ = 0.02. Overall, learners performed worse in the delayed test, *F*(1,174) = 34.91, *p* < 0.001, η^2^ = 0.17. An interaction between the condition and retention interval was found, *F*(1,174) = 7.93, *p* = 0.005, η^2^ = 0.04. Less forgetting occurred over a 2-week delay in the generation condition compared to students who read the cohesive text. The significant difference that was found between the conditions at T1 was not significant at T2 (*B* = 2.08, *t*(174) = 2.74, *p* = 0.007, 95% *CI* [0.58, 3.57], η^2^ = 0.04 vs. *B* = 0.49, *t*(174) = 0.72, *p* = 0.471, 95% *CI* [-0.86, 1.84] η^2^ = 0.00). No interaction between condition and reading skills was found, *F*(1,174) = 0.81, *p* = 0.369, η^2^ = 0.00.

#### Situation Model

**FIGURE 2:**
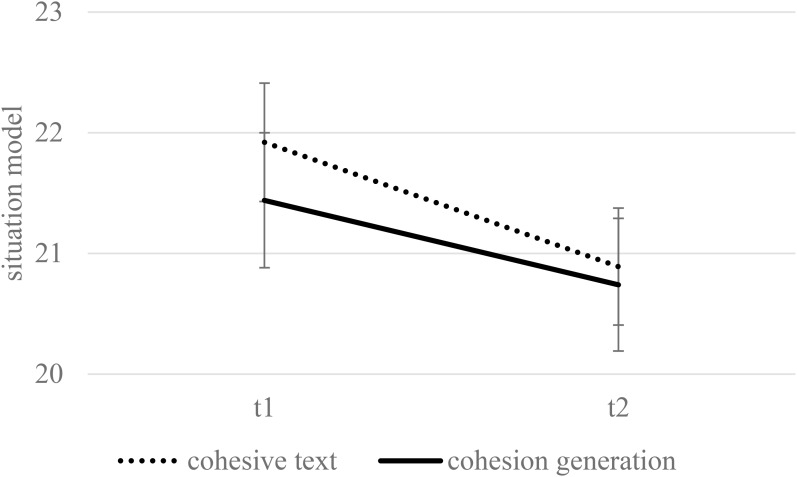
The situation model as a function of condition and delay when controlling for reading skills (estimated means and standard errors). Maximum performance was 42.

Figure 2 displays the means and standard errors for the situation-model questions in the final test as a function of condition and retention interval. No main effect of condition could be found, *F*(1,174) = 0.22, *p* = 0.641, η^2^ = 0.00, nor an interaction of condition and retention interval, *F*(1,174) = 0.30, *p* = 0.585, η^2^ = 0.00. Again, students performed worse during the delayed test, *F*(1,174) = 8.65, *p* = 0.004, η^2^ = 0.05.

**FIGURE 3:**
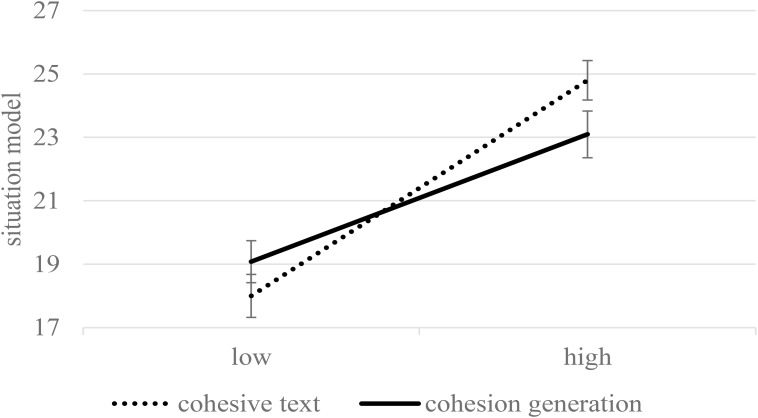
The situation model (collapsed across immediate and delayed testing) as a function of condition and the level of reading skills (estimated means and standard errors for –1 SD and +1 SD). Maximum performance was 42.

Reading skills had a significant impact on comprehension, *F*(1,174) = 64.25, *p* < 0.001, η^2^ = 0.27. More importantly, the impact of condition was moderated by learners’ reading skill level, *F*(1,174) = 4.27, *p* = 0.040, η^2^ = 0.02. Figure 3 displays the estimates for collapsed performance across T1 and T2 on the situation-model questions for learners with a high (+1 *SD*) and a low level of reading skills (-1 *SD*). Neither high-skilled readers scored significantly higher when reading the cohesive text, *p* = 0.077, nor low-skilled readers performed significantly better when generating cohesion, *p* = 0.258. No further significant interactions with reading skills were found.

### Learning Outcomes of Successful Generators

Given the high impact of generation success on learning, the relative benefits for students who performed highly accurately on the generation task compared to students who simply read the text was further explored. We repeated the analysis on text-based representation and the situation model by means of a repeated-measures ANCOVA, with condition as a between-subjects factor, delay as a within-subjects factor, and reading skills as a covariate. Only students with a generation success of ≥ +1 *SD* (*n* = 13) were included in the generation condition. These students generated ≥ 90% of causal relations correctly.

#### Text-Based Representation Analysis of Successful Generators

**FIGURE 4:**
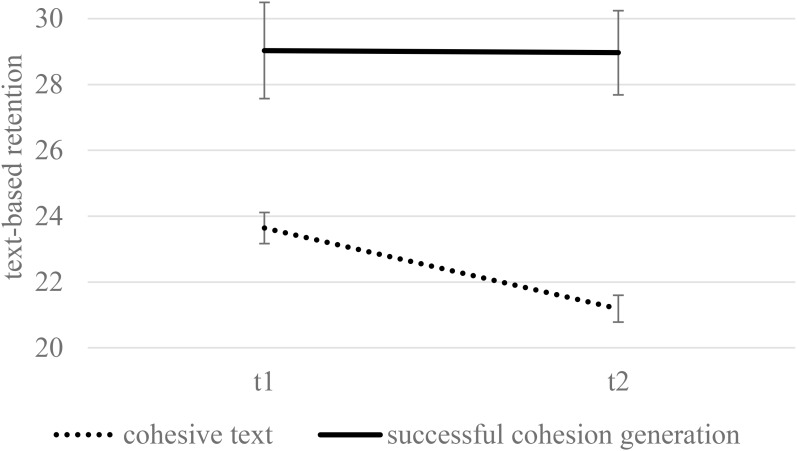
Text-based representation as a function of condition and delay when controlling for reading skills (estimated means and standard errors). In contrast to Figure 1, only learners who successfully generated (+1 SD) were analyzed. Maximum performance was 38.

**FIGURE 5:**
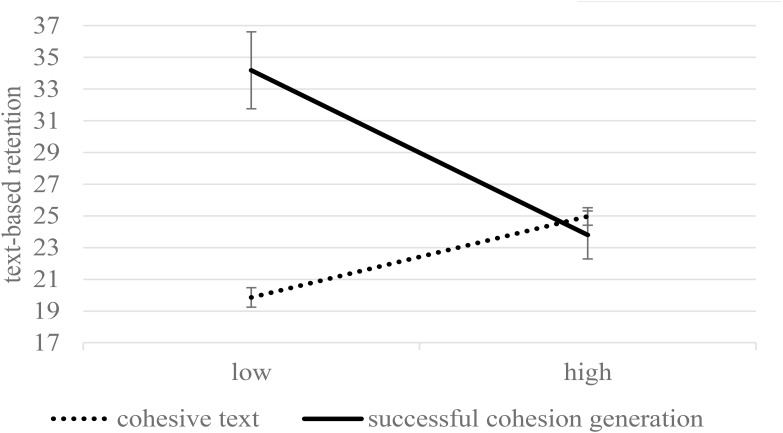
Text-based representation (collapsed across immediate and delayed testing) as a function of condition and the level of reading skill (estimated means and standard errors for –1 SD and +1 SD). Only learners who successfully generated (+1 SD) were analyzed. Maximum performance was 38.

The results of text-based representation analysis are depicted in Figure 4 (see Figure 1 for comparison with the entire generation group). Students who successfully performed on the generation task highly outperformed the students in the control condition, *F*(1,109) = 25.60, *p* < 0.001, η^2^ = 0.19. The performance decreased after the delay, *F*(1,109) = 4.14, *p* = 0.044, η^2^ = 0.04. The ANCOVA did not reveal a significant interaction of condition and delay, *F*(1,109) = 3.71, *p* = 0.057, η^2^ = 0.03. However, students in the generation condition showed less forgetting. Although the performance in the control condition decreased significantly, performance in the generation condition did not differ between immediate and delayed testing (*p* < 0.001, 95% *CI* [1.70, 3.20] vs. *p* = 0.955, 95% *CI* [-2.27, 2.40]). We also found an interaction effect between condition and reading skills, *F*(1,109) = 21.75, *p* < 0.001, η^2^ = 0.17. Figure 5 displays the estimates for collapsed performance across T1 and T2 for learners with a high (+1 *SD*) and low level of reading skill (-1 *SD*). Simple comparisons revealed no significant differences between condition for high-skilled readers (*p* = 0.473). However, the low-skilled readers showed superior learning performance in the generation condition compared to the control condition (*p* < 0.001). Thus, poor readers who achieved a high generation accuracy were greatly advantaged by the generation activity, but for skilled readers, the condition did not matter.

#### Situation Model of Successful Generators

**FIGURE 6:**
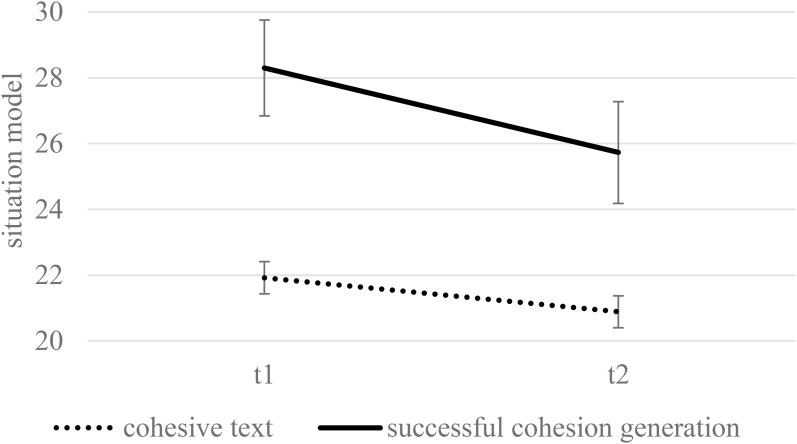
Situation model as a function of condition and delay when controlling for reading skills (estimated means and standard errors). In contrast to Figure 2, only learners who successfully generated (+1 SD) were analyzed. Maximum performance was 42.

**FIGURE 7:**
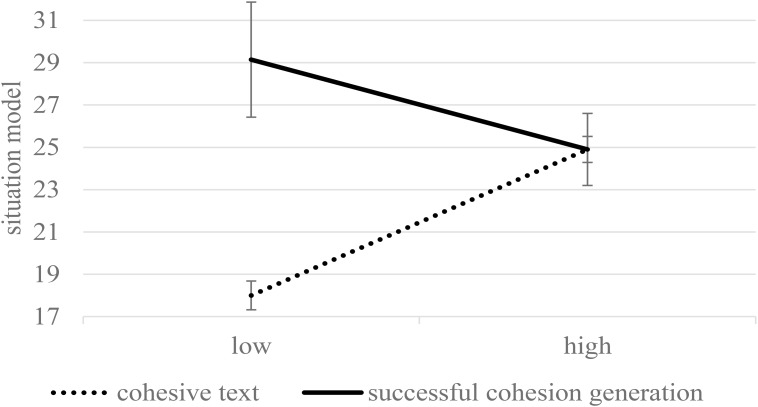
The situation model (collapsed across immediate and delayed testing) as a function of condition and the level of reading skill (estimated means and standard errors for –1 SD and +1 SD). In contrast to Figure 3, only learners who successfully generated (+1 SD) were analyzed. Maximum performance was 42.

The results of the situation model are presented in Figure 6 (see Figure 2 for comparison with the entire generation group). Students who successfully performed the generation task outperformed the students in the control condition, *F*(1,109) = 14.88, *p* < 0.001, η^2^ = 0.12. The performance decreased after the delay, *F*(1,109) = 8.35, *p* = 0.005, η^2^ = 0.07. No significant interaction of condition and delay was found, *F*(1,109) = 1.53, *p* = 0.218, η^2^ = 0.01. We further found an interaction effect between condition and reading skills, *F*(1,109) = 8.89, *p* = 0.004, η^2^ = 0.07. Figure 7 displays the estimates for collapsed performance across T1 and T2 for learners with a high (+1 *SD*) and a low level of reading skills (-1 *SD*; see Figure 3 for comparison with the entire generation group). Simple comparisons revealed no differences between the conditions in high-skilled readers (*p* = 0.968). In contrast, the low-skilled readers showed superior learning performance in the generation condition compared to the control condition (*p* < 0.001). Thus, poor readers who achieved a high generation accuracy were greatly advantaged by the generation activity, but for skilled readers, the condition did not matter.

## Discussion

The present study investigated the effect of causal-relation generation—as an innovative generative learning technique—on learning scientific content in high school. We compared students who generated cohesion connectives to students who read a fully cohesive text on learning processes and learning outcomes assessed by an immediate and a 2-week delayed test. We could not confirm our assumptions about the effects of generation on learning. We found no main effect of condition on situation-model construction, which contradicted H1. We also found that the immediate text-based representation was inferior to reading a fully cohesive text, contradicting H2. However, we found support for the remaining hypotheses. Students in the generation condition showed less forgetting, confirming H3. Generation success was highly predictive of the situation model even when controlling for learners’ proficiencies, confirming H4, but generation success was also a significant predictor of text-based representation. We predicted that text-based representation would be less dependent on generation success, resulting in learning benefits even for worse performers. Thus, the text-based results are not in line with H4. The effect of the learning condition on the situation model was moderated by reading skills, confirming H5. We discuss the results in terms of the conditions under which the generation of causal relations is an undesirable and when it is a desirable difficulty in learning.

In most learners, cohesion generation imposed extraneous cognitive load, resulting in inferior learning. However, the small group of learners who successfully performed well during the generation task took great advantage of generative activity. These advanced learners showed a superior performance on the situation model and text-based questions compared to learners who read a fully cohesive text. Their retention performance was shown to be more sustainable over time. Low-skilled readers especially gained an advantage from successful generation.

### When Is Generation Undesirable?

The generation task was implemented by means of cohesion gaps within the sentences. Learners were required to choose the appropriate causal connective to complete a sentence. To establish a correct causal relation between two propositions, learners were required to reflect on the relations among concepts in the text. The impact of generation on learning was expected to be particularly apparent in terms of situation-model construction (H1). The situation model is usually assessed with questions requiring the reader to connect multiple sentences. Thus, learners were expected to apply mental procedures to answer questions on the situation model which overlapped with mental procedures necessary to establish an appropriate causal relation during reading (cf. McNamara and Healy, 2000). However, the ANCOVA revealed no main effect of condition on the situation model for the total sample, irrespective of learners’ generation success.

The potential learning advantages of generation could have been reduced by the relatively low generation success.^2^ Many learners in the generation condition were unsuccessful in establishing coherence. However, generation is likely to unfold its potential only if learners perform the generation task successfully. This interpretation is supported by a strong correlation between generation accuracy and performance in response to the situation-model questions in the immediate and delayed tests. The predictive power of generation success remained significant even when controlling for learners’ proficiencies.

Apart from its impact on coherence formation, we also expected the generation condition to improve the text-based representation (H2). The generation task solely targeted the relation among factual statements. However, participants were required to reinstate the factual information and to check the adequacy of the generated solution to be able to conclude an appropriate relation among statements (Donaldson and Bass, 1980). Thus, the learning advantage of generation was assumed to involve learning content that also served as cues during the generation activity (Greenwald and Johnson, 1989), but participants in the present study who read the fully cohesive text outperformed participants who generated causal relations with respect to the text-based representation when immediately tested.

The text-based representation was expected to be less dependent on generation success than the situation model, because the text-based questions require only factual knowledge. Thus, the text-based representation was expected to be facilitated regardless of whether the sentences were correctly connected or not. Contrary to our expectation, generation success was significantly predictive of the text-based representation and explained a significant amount of variance when controlling for learners’ proficiencies. Participants who correctly determined causal relations between factual statements were likely to recall the factual information, probably because a full comprehension of factual statements is necessary to determine the nature of causal relations.

We speculate from this pattern of results that many learners were overwhelmed by the requirements of the generation task and sought syntactical and semantical hints rather than focus on meaningful aspects. The possibility of making inferences based on syntactical structures could have averted learners’ attention on meaning. According to the randomness as genesis principle, students without relevant schemas of how to perform the generation task rely on basic operations such as trial-and-error (Chen et al., 2015, 2016). The generation task was intended to widen attentional focus. However, many learners narrowed their attentional focus and processed the learning content fragmentally. Thus, learners who paid attention to irrelevant information units had not managed to construct a coherent or a basic representation of factual statements, resulting in verification of incorrect statements that include terms from the text and resulted in disaffirmation of correct statements that were slightly rephrased. The assumption about fragmented processing in many learners is supported by a higher extraneous cognitive load in the generation condition. A high difficulty of processing a generation task was indicated by a negative correlation between generation success and response reaction times during the dual task: Participants who experienced less cognitive load on working memory capacity could devote free cognitive resources to perform the generation task correctly. This interpretation is also supported by the positive correlation between generation success and germane load, and the negative correlation of generation success with extraneous load.

### Long-Term Retention

Confirming H3, participants in the generation condition forgot less after a 2-week delay with respect to the text-based representation, regardless of their generation success. The generation task may have reinforced the processing of single sentences. However, a lower forgetting rate in the generation condition did not result in higher text-based scores compared to the cohesive-text condition in the delayed test. The long-term advantage with text-based representations was especially clear in students who performed accurately during the generation task. The advantage of generation on text-based representations, compared to reading a cohesive text, was shown for the immediate testing. This advantage increased 2 weeks later because of the steeper forgetting rate in the cohesive-text group. These findings are consistent with the well-grounded generation effect in delayed tests (for a brief overview, see Chen et al., 2016). Furthermore, the meta-analysis by Bertsch et al. (2007) revealed an increase in effect sizes of generation benefits from immediate testing to more than a one-day delay.

In view of these findings and the current results, generation slows down forgetting. The effect was clearly pronounced in learners with high generation success, which suggests that the decreased rate of forgetting depends on deep processing. Elaboration of causal relations produces additional retrieval routes in memory, which in turn enhances retrieval (O’Brien and Myers, 1985). Numerous studies on learning techniques, which are considered to be desirable difficulties, revealed lower forgetting rates compared to conventional learning methods (e.g., rereading; for disfluency, see Weissgerber and Reinhard, 2017; for spacing and retrieval practice, see Delaney et al., 2010). These learning techniques were shown to slow down initial learning but to advantage learning in the long run (Richland et al., 2005).

### For Whom Is Generation Desirable?

High scores on generation accuracy led to a higher and more sustainable learning performance even when controlling for learners’ proficiencies (confirming H4). Thus, only accurate performers in the generation task greatly outperformed the students in the control condition in terms of the situation model and text-based retention.

Although generation success depended on learners’ proficiencies, such as reading skill, prior knowledge, and word analogy, high-skilled readers did not benefit from generative activity. In fact, skilled readers showed a more elaborated situation model after reading a fully cohesive text. No benefits of generation could be found even when only skilled readers who performed accurately on the generation task were analyzed. Skilled readers appear to spontaneously make use of explicitly marked links in text by generating world knowledge inferences (cf. Cozijn, 2000), and they exert more effort in achieving explanatory coherence (Magliano and Millis, 2003). Thus, generative activity might be redundant. In short, skilled learners are able to successfully generate cohesion, but they do not need it because of their ability to spontaneously engage in bridging inferences.

The impact of learning condition was different for high- and low-skilled readers (confirming H5). Remarkably, poor readers relied less than skilled readers on the instructional support provided by cohesion devices with reference to situation-model construction. When analyzing only students who performed accurately on the generation task, poor readers were greatly advantaged by the generation activity for both the text-based representation and situation model. This pattern of results can be attributed to the lack of spontaneous inferential processing in poor readers (McDaniel et al., 2002; McDaniel and Butler, 2011). In a complementary way, *explicitly* marked cohesion gaps engaged poor readers in inferential processing by minimizing the demands of detecting those gaps. In short, poor readers need stimulation provided by generative prompts, but they are less capable of performing accurately in the generation task. Consequently, poor readers require support on generating causal relations to unfold the full potential of generation.

### Limitations

One method limitation that needs to be addressed is our restricted selection of causal connectives that systematically varied along the two dimensions of direction and polarity. Other types of cohesion, such as the referential cohesion (Graesser et al., 2011), and other types of connectives, such as additive or temporal (Louwerse, 2001), and the specialization in either objective (consequence-cause) or subjective (claim-reason) causal relations (Traxler et al., 1997; Canestrelli et al., 2013) were omitted. In follow-up studies, the generation task could be implemented either by forced choice between certain types of connectives or by using a free generation format in which participants could fill the gaps without any restrictions.

From another perspective, our restriction of using only causal connectives can be considered a strength of our method for three reasons. First, the research on how text characteristics affect learning can be differentiated by the broadness of the to-be-manipulated text characteristics and the length and complexity of texts (Van Silfhout et al., 2014a). Many studies have manipulated a very narrow text characteristic (e.g., whether *because* occurs or not) using isolated sentences or short texts. In contrast, other studies have defined cohesion broadly, varying many characteristics at once throughout full-length texts. We advanced the research by simultaneously manipulating just one text characteristic in full-length expository text. Second, deep understanding of scientific phenomena, such as the greenhouse effect and climate change, requires learners to understand causes and consequences in dynamic systems. Understanding causal relations should therefore be the major aim of studying such phenomena. Third, additional types of connectives, such as additive and temporal, are underspecified if they serve in causal relations (Sanders and Noordman, 2000; Louwerse, 2001) and less important for understanding (Noordman and Vonk, 1997). In other words, temporal and additive connectives provide no additional information that is not already addressed by causal connectives in causal relations. Instead, causal relations typically imply temporal and additive relations. In line with this reasoning, Goldman and Murray (1992) found that students overuse causal connectives compared to other types of connectives.

We used a dual task to objectively measure the cognitive load imposed by the generative activity. A dual task usually serves one of two possible functions by either interfering with the learning activity, which consumes necessary cognitive resources (time-on task and accuracy on the prior task would indicate the degree of interference), or the task is affected by the learning activity. An aim of the present study was to measure the impact of the generation task on cognitive processes. The dual task had a very low level of difficulty and thus did not resemble the requirements of text comprehension. We therefore expected the generative activity to be unaffected by the dual task. However, the possibility of posing additional load on learners’ working memory and interfering with the generation task cannot be excluded (cf. Brünken et al., 2002).

We manipulated learning performance as a within-subjects factor (i.e., students were tested immediately and after a 2-week delay), because we were particularly interested in how generative learning affects forgetting rates. This method poses a possible limitation of the effect. Generation effects from T1 to T2 could have been confounded by the testing effect (cf. Butler, 2010). Learners who read a fully cohesive text could especially gain an advantage from being required to retrieve learning content by retention-based items and to elaborate on the learning content by inference-based items (Roelle and Berthold, 2017).

### Issues for Implementation and Future Directions

In the present study, we attempted to promote relational processing by requiring students to generate causal relations during reading. To provide teachers and learners with an innovative learning technique, the implementation of the generative activity was intended to be easily applicable in educational settings (Dunlosky et al., 2013). Filling gaps in incomplete sentences is known as a conventional way to promote active processing in school. Thus, students are familiar with this type of task, commonly called *fill-in-the-blank*. Accordingly, choosing the appropriate term might be free from the extraneous load associated with unfamiliarity of the task type.

Although the type of task resembles the well-known fill-in-the-blank technique, the causal cohesion may have appeared to students as an unusual generative activity. Generally, students are inexperienced in reflecting causal relations in terms of direction and polarity. Students especially struggled to correctly determine a negative causal relation, reflecting higher cognitive demands to process adversative causal relations (Goldman and Murray, 1992; Knoepke et al., 2016). Students in our study were challenged by the cognitive demands imposed by an unusual generation target. A relatively low generation success and high scores in cognitive load measures support this view. A consequence of method unfamiliarity could have resulted in an overestimation of the positive impact when reading the fully cohesive text. The potential of the cohesion generation task may have been suppressed by the learners’ inexperience with this method (cf. Rummer et al., 2017). To allow for a *fair* comparison between the effectiveness of a fully cohesive text and generating cohesion during reading, familiarity with the activity should be similar between conditions. Familiarity of generative activity notwithstanding, an accurate performance during the generation task is crucial for learning, and students rely heavily on support to perform the generation task.

In follow-up studies on cohesion generation, instructional support in combination with a practice phase should compensate for the inexperience with a generative learning strategy. The instruction should steer learners’ attentional focus to the dimensions of causality, namely direction and polarity. Identifying the correct connective in a given constellation of factual statements requires learners to address the following questions: *Which fact is the cause and which is the consequence?* *Have I expected that A follows from B, or does their relation contradict my expectation?* Selecting the connective *because*, *therefore*, *although*, or *however* directly depends on the answers to these two questions. That is, learners must systematically apply this knowledge to correctly determine the appropriate causal relation. Practicing cohesion generation with corrective feedback might therefore reinforce the autonomous use of this knowledge of causal cohesion during generative learning. Recent evidence points to the advantages of instructional support in increasing sensitivity to causal patterns (Goldwater and Gentner, 2015) or identifying structural components of arguments (Von der Mühlen et al., 2018). Short-term training might particularly increase the awareness in learners about the appropriateness of using cohesion devices.

One further possibility of improving learners’ generation performance is to provide them with corrective feedback on their lexical decisions, which was not employed in the present study. For example, in the follow-up study, students could read the text two times. In the generation condition, students could perform the generation task during the first reading, then after receiving the fully cohesive text to be able to reflect on their lexical decisions.

## Final Conclusion

Generation is considered a desirable difficulty in learning (Bjork and Bjork, 2014). However, three conditions must be fulfilled to make a difficulty desirable. First, difficulty should promote the processes required to answer questions in the final test (McDaniel and Masson, 1985; McNamara and Healy, 2000). Second, difficulty should promote the processes of knowledge construction not spontaneously initiated by learners (McDaniel and Butler, 2011). Third, difficulty should be surmountable for learners (O’Brien and Myers, 1985). In this study, we proposed an innovative generative learning technique for educational practice. Generation of causal relations appears to be a promising learning tool, because it already fulfills two of the three conditions. First, to establish a coherent mental representation of the text, learners are required to infer the causal relations among the factual statements (process of organization) and to integrate the factual statements with previous knowledge by making world knowledge inferences (cf. Cozijn et al., 2011). As a result, a coherent mental representation supports learners’ ability to make inferences required by the final test. Second, cohesion generation can benefit poor readers, because poor readers are usually not engaged in the spontaneous processes of organization and integration (cf. Fiorella and Mayer, 2016). The third condition, however, was not met in this study. The necessary support to overcome the difficulty imposed by the generation task was not provided. Nonetheless, learners who succeeded to employ effortful processing to overcome the difficulty, took great advantages of generative activity. Future research on cohesion generation should incorporate instructional support on the meaning of the two causality dimensions, direction and polarity (Sanders et al., 1992; Louwerse, 2001), including an opportunity to practice. We look forward to further discoveries in the effects of cohesion generation on long-term retention and coherence construction by boosting the generation success rates.

## Ethics Statement

This study was carried out in permission and in accordance with the recommendations of Hessian Ministry of Culture. We received parental consent for all participants under 18.

## Author Contributions

RA and MH contributed to conception and design of the study. RA organized the database, performed the statistical analysis, and wrote the manuscript. MH contributed to the manuscript revision and read and approved the submitted version.

## Conflict of Interest Statement

The authors declare that the research was conducted in the absence of any commercial or financial relationships that could be construed as a potential conflict of interest.

## Footnotes

1 We also analyzed whether certain connectives were chosen with a higher accuracy relative to other ones. If so, the second aim would be to determine the impact of the connectives’ dimensions of causality direction and polarity on generation accuracy. We computed the accuracy rates of all four connectives. An ANOVA with two within-subject factors for direction (forward vs. backward) and polarity (positive vs. negative) was computed. The polarity of causal relations was found to have the highest impact on generation accuracy [*F*(1,86) = 144.77, *p* < 0.001, η^2^ = 0.63). Namely, negative connectives, however (*M* = 59.20, *SD* = 26.72), and although (*M* = 53.69, *SD* = 21.34) were more difficult to correctly identify compared to positive connectives therefore (*M* = 80.19, *SD* = 17.76) and because (*M* = 80.14, *SD* = 17.60), indicating a higher cognitive demand of encoding negative causal relations. We found no significant main effect for direction, *F*(1,86) = 3.75, *p* = 0.056, η^2^ = 0.04, nor a significant interaction between direction and polarity, *F*(1,86) = 2.90, *p* = 0.092, η^2^ = 0.03.

2 The average rate of success was 74%. Given the unequal distribution of different connectives across the text and the presence of syntactical hints that indicated the direction of causal relations (see learning materials), the mean error rate of 26% can be regarded as relatively high.
